# Supplementary figures and images for: Feasibility, Reproducibility and Reference Ranges of Left Atrial Strain in Preterm and Term Neonates in the First 48 h of Life
Source: Diagnostics (Basel). 2022 Jan 29;12(2):350. doi: 10.3390/diagnostics12020350 (PMC8871374; doi:10.3390/diagnostics12020350)

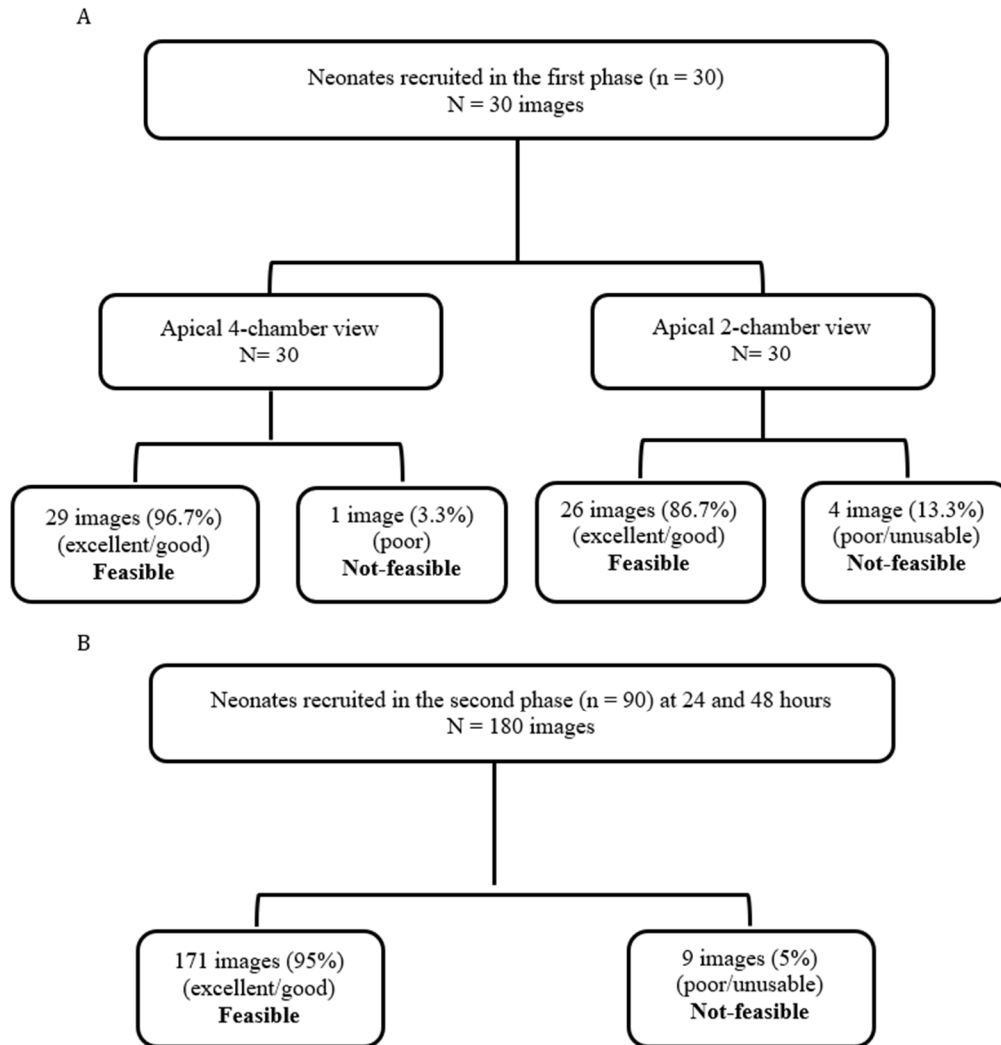

**Figure S1.** Feasibility flow chart for (A) the first phase and (B) the second phase of the study.

Supplement: Supplementary file 1 [file diagnostics-12-00350-s001.zip › diagnostics-1512358-supplementary.pdf]
